# Flow-dependent fluorescence of CCVJ

**DOI:** 10.1186/s13036-017-0067-2

**Published:** 2017-08-03

**Authors:** Markus J. Schmidt, David Sauter, Thomas Rösgen

**Affiliations:** Institute of Fluid Dynamics, ETH Zurich, Zurich, 8092 Switzerland

**Keywords:** Fluid dynamics, Fluid mechanics, Molecular rotors, Flow sensors, Viscosity, Shear stress, Microfluidics, Photoisomerization, Molecular tagging velocimetry, Twisted intramolecular charge transfer states, TICT

## Abstract

**Background:**

The molecular rotor 9-(2-Carboxy-2-cyanovinyl)julolidine (CCVJ) is presumed to have a sensitivity towards velocity or shear which is supposed to result in a change in fluorescence quantum yield. Furthermore, a previously reported photoisomeric behavior may contribute to the measured fluorescence intensity changes. The goal of this research was to examine the hypothesized behavior theoretically and experimentally from the perspective of fluid dynamics.

**Results:**

A correlation between stirring rate and intensity could not be established in the present experiments with a completely illuminated sample in contrast to previously reported experiments in spectrofluorometers. Experiments and theoretical models of a Poiseuille flow were in good agreement with the photoisomeric behavior but excluded the influence of shear. Further experiments in a flow chamber supported the photoisomery hypothesis as well.

**Conclusion:**

No experimental evidence for the influence of velocity on the fluorescence intensity of CCVJ was found. The hypothesis of shear sensitivity was excluded as well. The results are consistent with the photoisomeric behavior of CCVJ.

## Background

In the field of microfluidics and the development of labs-on-a-chip, the experimental validation of the established fluid flow is a crucial point [1]. Non-intrusive methods are preferred for these validations, since the influence of intrusive methods on the flow may become significant due to the small spatial scales involved. As a specific example, molecular rotors are in use for the measurement of fluid viscosity [2]. Recently, a velocity or shear sensitivity of the molecular rotor 9-(2-Carboxy-2-cyanovinyl)julolidine (CCVJ) has been proposed.

### Observations of altered intensity in fluid flow

First observations of an intensity change were made by Haidekker et al. [3]. When a solution of CCVJ was stirred in a spectrofluorometer, a change in intensity was noted depending on the stirring rate of the magnetic stirrer. In addition, the measurement of a Poiseuille flow provided a changing intensity depending on the volume flow rate. The fluorescence signal was measured with an optical fiber aligned in the flow direction for both excitation and emission acquisition. The result led to the supposition of a relation between flow velocity and emission intensity. Based on their results, empirical fits were formulated ([3], Eqs. (2) and (4)):

1 $$\begin{array}{*{20}l} \!\!\!\!\!\Delta I &= \Delta I_{max} \left[ 1-\exp\left(-\frac{v}{v_{c}}\right)\right]  \end{array} $$

2 $$\begin{array}{*{20}l} \!\!\!\!\!\Delta I &= \Delta I_{max} \left[ 1-\exp\left(-\frac{v}{v_{c}}\right)\right] \cdot \left[ 1-\exp\left(-\frac{\eta}{\eta_{c}}\right)\right]  \end{array} $$

with *Δ* *I* being the intensity change, *Δ* *I*_*max*_ its maximum, *v* the flow velocity, *η* the fluid viscosity and *v*_*c*_, *η*_*c*_ as characteristic scaling constants for flow velocity and fluid viscosity, respectively. In this context, *Δ* *I* is supposed to be positive which results in an intensity increase.

Furthermore, a relation with the shear in the fluid flow was suggested. “The additional effect of fluid shear stress on this complex interaction is widely unknown, but it is conceivable that fluid flow alters the reorientation due to the much higher internal friction forces of the fluid.” (p. 48). In this context, the stirring rate is related to shear in the flow. As potential physical reason the authors mentioned a polar-solvent interaction [3].

The research was extended by another study [4]. The fluid flow was examined in different flow chambers. Besides fluorescence imaging, particle image velocimetry (PIV) experiments and computational fluid dynamics (CFD) simulations were conducted to support the argument of a sensitivity of CCVJ towards fluid flow properties. In addition, stirring experiments were conducted similar to [3] which confirmed the observations made. The study was not in complete agreement with the stated hypotheses. In particular, the phenomenon was predominantly noticed at the inlets of the flow chambers and not at the outlets [4].

### Photoisomerization of CCVJ

Rumble et al. found a different possible explanation for the intensity variations [5]. Their research came to the conclusion that a photoisomeric reaction of CCVJ may be responsible for the observed changes in fluorescence. Without illumination, CCVJ is present as E isomer. When illuminated, it converts to the nonluminescent Z isomer. Furthermore, they conducted syringe flow experiments and observed a relation between flow velocity and intensity. They hypothesized that the change in intensity is related to photoisomerization [5].

Considering the aspect of photoisomerization, Mustafic et al. conducted further experiments with CCVJ and other species [6]. They acknowledged the effect of photoisomerization present in observations of CCVJ. In addition, they stated that an additional effect is observable.

The results showed a relation between flow velocity and emission intensity as in the preceding studies. Due to the syringe flow experiments an additional effect to photoisomerization was anticipated. The assumption of a shear sensitivity, as stated in the title of the article, was not validated experimentally. The question of another measurable effect with CCVJ remained unanswered [6].

### Further research

Beyond the aforementioned discussion, CCVJ has been investigated by several other authors. Akers et al. [7] investigated the binding affinities of CCVJ and three of its derivatives regarding blood plasma proteins. In addition, the effects of solvent polarity and viscosity of various molecular rotors were experimentally examined by Haidekker et al. [8], where a CCVJ derivative was subject of the study.

The fluorescence anisotropy of CCVJ and DCVJ was investigated by Levitt et al. [9]. It has to be noted that no data was provided for CCVJ for the results of the steady-state anisotropy. Further research would be preferable to check whether the missing result can be explained photoisomerization.

In addition, the fluorescence lifetime of CCVJ was investigated under consideration of several cyclodextrines present in the solution [10]. The study claimed an increased quantum yield due to the use of those chemical supramolecules and provided evidence for the isomerization and its hindrance due to complex formation.

Recently, CCVJ and a newly developed derivate were applied in measurements of viscosity in membranes [11]. A remarkable result was the significantly higher fluorescence quantum yield of this derivative. A discussion of photoisomerization is missing, which could provide a possible explanation for the different quantum yields.

### CCVJ behavior in fluid flow

With the title “Apparent Shear Sensitivity of Molecular Rotors in Various Solvents” Mustafic et al. [6] suggested a shear sensitivity of molecular rotors such as CCVJ. They concluded: “We have found additional evidence that the recently discovered formation of a photoisomer plays an important role in the flow response, but E-Z isomerization alone cannot explain all observations” ([6], p.738). The question to be addressed within this work is the existence of a fluid flow or shear sensitivity of CCVJ in the controlled presence of photoisomerization.

First, experiments of a CCVJ-doped fluid flow in a spectrofluorometer, whether initiated with a stirring bar or a syringe pump, resulted in a change of the fluorescence emission intensity compared to the fluid at rest [3–6]. A proposed explanation for this effect is a flow velocity or shear sensitivity of the applied molecular rotor CCVJ. Those experiments are the strongest indication of an additional effect. Since the results of a syringe flow in a spectrofluorometer were assigned to isomerization by Rumble et al. [5] and Mustafic et al. [6], the motivation to conduct a different stirring experiment is to avoid the isomerization process present in these experiments. Hence a different setup is chosen.

Second, a similar relation was found conducting Poiseuille flow measurements by Haidekker et al. [3] These experiments are the foundation of the empirical formulae (1) and (2). The question remains if the results can be related to photoisomerization.

Third, the flow chambers examined by Mustafic et al. [4] were solely analyzed under the hypothesis of velocity and shear sensitivity. Further investigation can shed light on the influence of photoisomerization on these measurements.

Another molecular rotor, 9-(2,2-Dicyanovinyl)julolidine (DCVJ), is closely related to CCVJ. The chemical structure of DCVJ only permits one isomer [5]. Hence it was used as a control compound in the mentioned experiments above [3–6, 9]. The present work also uses DCVJ for comparison of the results.

In experimental fluid dynamics, measurement methods such as the use of photobleaching [12] and photochromic dyes [13] are in effect similar to the reported photoisomeric behavior. In experimental fluid dynamics, Particle Image Velocimetry and Molecular Tagging Velocimetry are established for the measurement of flow velocities [14, 15]. The goal of the present work is to further examine the behavior of CCVJ from a fluid dynamical perspective with a focus on the proposed existence of an additional effect next to photoisomerization and its applicability to fluid dynamics in the defined three topical areas.

## Methods

The discussion to follow is separated into three parts. First, a section on theory and analytical models as well as post-processing used in this work is presented. Second, a description of the fluorescent solution preparation follows. Third, different experimental setups, including a stirring experiment, flow chamber production and setup for further experiments with different excitation settings are introduced.

### Theory and analytical models

#### Intensity decay in a rectangular Poiseuille flow

Related to the experiment with a rectangular Poiseuille flow, the analytical solution is complemented with a fluorescence decay to model a possible effect of photoisomerization. The analytical solution of the Navier Stokes equation for the velocity field in a fully developed rectangular Poiseuille flow is given by

3 $$  \begin{aligned} u (y,z) &= \frac{1}{\eta}\frac{\partial p}{\partial x} \left[\frac{1}{2}\left(y^{2} - \frac{h^{2}}{4}\right) - 4h^{2} \sum_{n=1}^{\infty} \frac{(-1)^{n}}{s_{n}^{3}}\right.\\ &\qquad\qquad\times\left.\frac{\cosh\left(s_{n}K\frac{z}{w}\right)}{\cosh\left(s_{n}K\frac{1}{2}\right)}\cos\left(s_{n} \frac{y}{h}\right)\right] \end{aligned}  $$

with *h* and *w* as the channel height and width, $\frac {\partial p}{\partial x}$ the pressure gradient in the flow direction, *η* the fluid dynamic viscosity, $K=\frac {w}{h}$ the cross-section aspect ratio and *s*_*n*_=(2*n*−1)*π* ([16], eq. (19)). The *x*, *y* and *z* directions describe the channel coordinates regarding length, height and width, respectively. The origin of the coordinate system is defined in the center of the channel. The pressure gradient can be estimated as

4 $$ \left| \frac{\partial p}{\partial x} \right| \approx \frac{\Delta p}{L} \approx \frac{Q}{1- 0.630 \frac{h}{w}}\cdot \frac{12\eta}{h^{3}w}  $$

with *Q* the volumetric flow rate and *L* the length of the channel ([17], eq. (63c)).

For the hypothesis of a photoisomeric effect, assuming a mono-exponential decay, the relative intensity change can be described as

5 $$  \delta I_{rp}(x,y,z) = \frac{I_{E,0}}{I_{base}} \cdot \exp\left(-\frac{t_{irr}(x,y,z)}{t_{decay}}\right)  $$

with *I*_*E*,0_ as the initial contribution of intensity of the E isomer, *t*_*decay*_ the characteristic decay time constant, assuming a constant irradiation, and *I*_*base*_ a baseline intensity to be chosen. The time a fluid element is exposed to light is given by

6 $$ t_{irr}(x,y,z) = \frac{x}{u(y,z)}  $$

An integration

7 $$ \overline{\delta I_{rp}}(x,y) = \frac{1}{L} \int\limits_{0}^{L} \delta I_{rp}(x,y,z) dz'  $$

can be applied to obtain a 2D projection of the 3D results for comparison with experimental results.

#### Fluorescence signal of CCVJ in an optical fiber

Haidekker et al. [3] examined the flow in a circular channel with a modified optical fiber and measured the intensity in the flow direction. A one-dimensional model of such a flow in combination with the photoisomeric behavior of CCVJ can be developed to address a possible relation of isomerization to the experimental results.

**Fig. 1:**
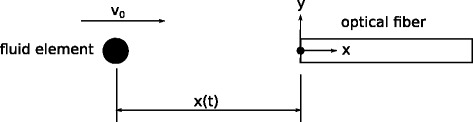
Model of a fluid element with CCVJ. The fluid element propagates toward the optical fiber with velocity *v*_0_ while illuminated

A schematic can be found in Fig. 1. An optical fiber, oriented in the counter-flow direction, emits light with a constant intensity *I*_*ex*,0_.

##### **Assumption 1**

The excitation light intensity of the fiber is absorbed by fluid elements containing CCVJ.

##### **Assumption 2**

The emitted fluorescence by CCVJ is not absorbed on its way to the fiber.

In the one-dimensional Lagrangian description, the light is absorbed by fluid elements within the path of light. This absorption leads to a decrease in fluorescence intensity which can be described with

8 $$ I_{ex}(x) = I_{ex,0} \cdot \exp\left(\varepsilon C_{tot} x\right)  $$

with *I*_*ex*,0_ the initial excitation light intensity of the fiber, *ε* the absorption coefficient and *C*_*tot*_ the total concentration of CCVJ. Note that in the present model the position of the fluid element is always left of the origin.

##### **Assumption 3**

The absorption of light does not depend on the isomer state of CCVJ, *C*_*tot*_=*c* *o* *n* *s* *t*.

The concentration of E isomer *C*_*E*_ can be computed in two different ways. The first approach starts with the reaction kinetics of the two species. The concentration can be described with the rate equation

9 $$ \frac{\partial C_{E}}{\partial t} = -\kappa_{EZ}\cdot C_{E} + \kappa_{ZE}\cdot C_{Z}  $$

with *C*_*E*_, *C*_*Z*_ as the concentration of the respective isomers and *κ*_*EZ*_, *κ*_*ZE*_ as the rate coefficients.

##### **Assumption 4**

The rate constant from Z to E isomer is much smaller than the reverse, *κ*_*ZE*_≪*κ*_*EZ*_.

##### **Assumption 5**

The rate constant *κ*_*EZ*_ is proportional to the excitation light intensity from the fiber, *κ*_*EZ*_=*k*_*abs*_·*I*_*ex*_(*x*).

##### **Assumption 6**

The position of a fluid element depends on time according to *x*(*t*)=*x*_0_+*v*_0_ *t*, with *x*_0_ an initial position and *v*_0_ the velocity of the element.

This leads to the solution

10 $$\begin{array}{*{20}l}  \frac{\partial C_{E}}{\partial t} &= -k_{abs}I_{ex}(x)\cdot C_{E} \end{array} $$

11 $$\begin{array}{*{20}l} \frac{\partial C_{E}}{\partial t} &= -k_{abs} I_{ex,0} \cdot \exp\left(\varepsilon C_{tot} x(t)\right) \cdot C_{E} \end{array} $$

12 $$\begin{array}{*{20}l} C_{E}(x) &= k_{1}\cdot\exp\left[-\frac{k_{abs}I_{ex,0}}{C_{tot}\varepsilon v_{0}}\exp\left(C_{tot}\varepsilon x\right)\right] \end{array} $$

with *k*_1_ an integration constant.

A second approach uses the material derivative of the concentration of the E isomer in the Eulerian description, where the phenomena are described as steady,  which also leads to the solution in Eq. (10). Note that in the Eulerian description the flow field is stationary.

##### **Assumption 7**

Far away from the illumination source, CCVJ is in an equilibrium with an initial concentration of E isomer *C*_*E*,*∞*_.

13 $$ C_{E}(-\infty) = C_{E,\infty} = k_{1}\cdot\exp(0)  $$

which leads to the solution for the E isomer concentration

14 $$ C_{E}(x) = C_{E,\infty}\cdot\exp\left[-\frac{k_{abs}I_{ex,0}}{C_{tot}\varepsilon v_{0}}\exp\left(C_{tot}\varepsilon x\right)\right]  $$

The fluorescence intensity acquired by the optical fiber depends on the absorbed light, the concentration of CCVJ and the quantum yield *Φ*_*fl*_ integrated over the line of illumination

15 $$\begin{array}{*{20}l} I_{fl} &= \int\limits_{-\infty}^{0}\Phi_{fl}\cdot C_{E} \cdot I_{ex} dx' \end{array} $$

16 $$\begin{array}{*{20}l} &= \left.-\frac{\Phi_{fl}C_{E,\infty}v_{0}}{k_{abs}}\exp\left[-\frac{k_{abs}I_{ex,0}}{C_{tot}\varepsilon v_{0}}\exp\left(C_{tot}\varepsilon x\right)\right]\right|_{-\infty}^{0} \end{array} $$

17 $$\begin{array}{*{20}l} &= \frac{\Phi_{fl}C_{E,\infty}v_{0}}{k_{abs}} \left\{1- \exp\left[-\frac{k_{abs}I_{ex,0}}{C_{tot}\varepsilon v_{0}}\right] \right\}  \end{array} $$

The computed intensity shows a dependency on the flow velocity *v*_0_. In the case of small velocities the intensity becomes linearly dependent on it

18 $$ v_{0} \ll \frac{C_{tot}\varepsilon}{k_{abs}I_{ex,0}} \rightarrow I_{fl} \propto \frac{\Phi_{fl}C_{E,\infty}v_{0}}{k_{abs}}  $$

#### Intensity computation

In a first step, the acquired images were normalized using a reference image to account for temporal variations in the light source emission. A target area is added to the image acquisition system with suitable reflectivity properties. From the acquired images, one image is chosen as reference image, typically the no-flow image. The target area of size *N*·*M* is then used to compute a weight factor for the intensity variation

19 $$\begin{array}{*{20}l} \overline{\delta I_{w}} = \frac{1}{NM}\sum_{x=1}^{N}\sum_{y=1}^{M} \frac{I_{ref}(x,y)}{I(x,y)} \end{array} $$

with *I*(*x*,*y*) the current image and *I*_*ref*_(*x*,*y*) the reference image. This leads to an intensity-corrected image

20 $$ I_{cor}(x,y) = \overline{\delta I_{w}} \cdot I(x,y)  $$

The relative intensity variation is computed with a normalized difference of a corrected flow *I*_*f*_(*x*,*y*) and a corrected no flow image *I*_*nf*_(*x*,*y*). For both conditions a number of *J* images are acquired and averaged

21 $$ \delta I(x,y) = \frac{1}{\overline{I_{nf}}\cdot J} \left[\sum_{i=1}^{J} I_{f,i}(x,y) - \sum_{i=1}^{J} I_{nf,i}(x,y)\right]  $$

with $\overline {I_{nf}}$ the mean pixel value of the no flow images in the region of interest (ROI).

### Solution preparation

CCVJ, DCVJ and dimethyl sulfoxide (DMSO) were purchased from Sigma-Aldrich. The dyes were dissolved in 2 mL DMSO. After preparation, the stock solutions were stored in the dark at room temperature. Mixture details can be found in Table 1.

**Table 1:** Preparation of stock solution

Dye	CCVJ	DCVJ
Dye Mass (mg)	2.4	2.2
Molecular Weight (g/mol)	268.31	249.31
Solvent (mL)	2.0	2.0
Dye Concentration (mol/L)	4.5·10^−3^	4.4·10^−3^

The solutions for the experiments were prepared with ethylene glycol (Sigma-Aldrich) and the concentration was chosen following Mustafic et al. [4] The compositions of the solutions are listed in Table 2.

**Table 2:** Preparation of experimental solutions with ethylene glycol

Dye	CCVJ	DCVJ
Stock Solution (mL)	0.675	1.37
Solvent (mL)	200	400
Dye Concentration (*μ*mol/L)	15.04	15.06

### Experimental setup for stirring

**Fig. 2:**
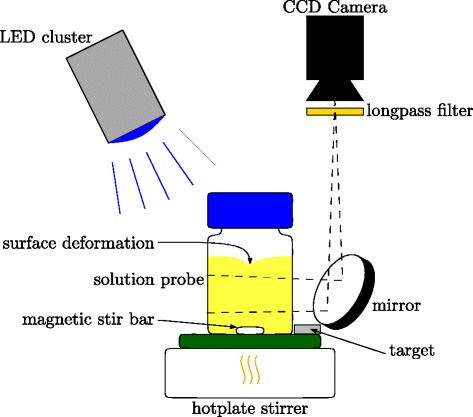
Setup for stirring experiments. The acquired image is not influenced by interferences of the magnetic stirrer or the fluid surface

For the examination of a possible stirring dependency of CCVJ fluorescence, a closed 100 mL laboratory bottle was placed on a hot plate stirrer. The setup is shown in Fig. 2. A magnetic stir bar was used for the stirring. A light emitting diode (LED) cluster with Sloan L5-B91G-WT LEDs was used for the illumination of the complete sample. The cluster consisted of 61 LEDs with a power of 120 mW each and a peak wavelength of 470 nm. A mirror was placed such that neither the magnetic stir bar nor the deformed fluid surface had an influence on the measurements. The light passed through a Schott OG 515 longpass filter and was acquired with a JAI RM-4200GM camera and a Nikon AF NIKKOR 50 mm 1:1.4D objective.

### Flow chambers manufacture

The dimensions of the flow chambers were taken from Mustafic et al. [4] In addition, a Poiseuille flow chamber was designed. Similar to the other flow chambers, the connections were round inlets with 5 mm diameter. The channel itself had a rectangular shape of 5 mm width, 3 mm depth and a length of 93 mm. The chamber is denoted with letter E. Figure 7 shows the newly designed chamber E, partly covered.

All chambers were produced from transparent, 12 mm thick acrylic. The chamber geometries were milled in one plate with a depth of 3 mm and a rubber gasket was mounted around the geometries for sealing. Furthermore, a black diffuse adhesive foil was placed on the chamber floors to suppress reflections and background observations. The foils were laser-cut for a precise fit.

### Experimental setup for the flow chambers

Each chamber was mounted below the image acquisition system, consisting of the aforementioned filter and camera. A Cavro XLP 6000 Modular Syringe Pump equipped with a 5-ml glass syringe and a two-way valve was used for establishment of the flow. The illumination and image acquisition timing were controlled by a Berkeley Nucleonics Model 500A pulse generator.

#### Continuous excitation

**Fig. 3:**
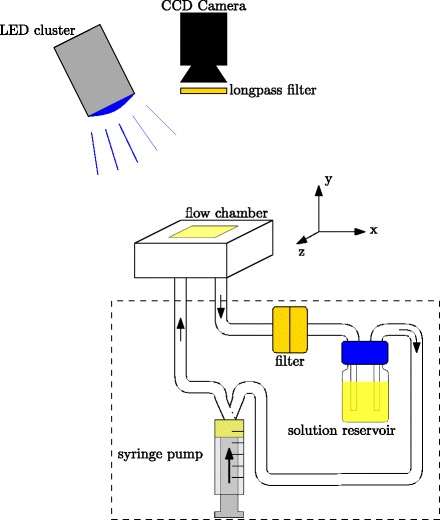
Setup for continuous excitation. A blue LED is used for continuous illumination. Components in the dashed rectangle were covered by black fabric

For experiments with continuous illumination, the blue LED cluster was used. The principal setup can be seen in Fig. 3. For these experiments all constructed flow chambers were separately examined, the chambers A to D as designed by Mustafic et al. [4] and the new Poiseuille flow chamber E. The LED was positioned to ensure a complete illumination of the flow chamber while not exciting the reservoir. The setup was held in darkness before starting the experiments. Furthermore, the reservoir and tubing were covered with fabric. Images with no flow and an established flow regime were taken subsequently.

#### Single shot excitation

**Fig. 4:**
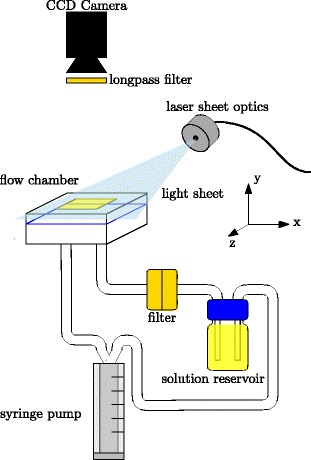
Setup for single-shot excitation. A blue laser is used for partial illumination of the test section

For single shot excitation, a Laser Quantum “Ciel” laser with a wavelength of 473 nm was connected to a light sheet optic. The laser was modulated to generate pulses of about 200 ms duration. The setup can be seen in Fig. 4. The laser sheet was aligned to illuminate the chamber in a plane perpendicular to the flow direction. Otherwise the setup was identical to the continuous excitation.

## Results

### Stirring

**Fig. 5:**
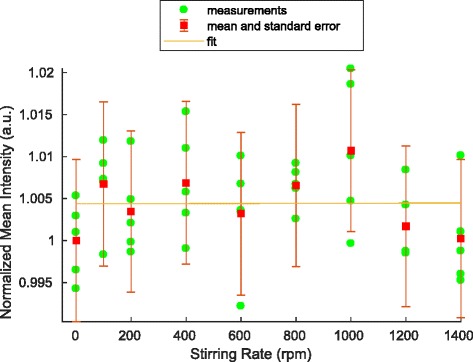
Stirring-intensity relation of CCVJ. Mean result of five acquisitions at each stirring rate (*green circles*), the mean and standard error of all pixels (*red*) and fit with *R* ^2^=3.7·10^(−3)^ (*orange line*)

**Fig. 6:**
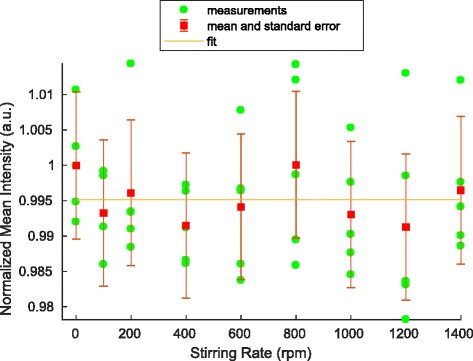
Stirring-intensity relation of DCVJ. Mean result of five acquisitions at each stirring rate (*green circles*), the mean and standard error of all pixels (*red*) and fit with *R* ^2^=−1.03·10^(−4)^ (*orange line*)

The laboratory bottle was exposed to the blue LED illumination for several minutes to ensure a chemical equilibrium in favor of the Z isomer. At each stirring rate, 5 images were acquired. Normalized intensity values in the region of interest are shown in Figs. 5 and 6 for CCVJ and DCVJ, respectively. The results are normalized by the mean intensity of the measurements at rest. A fit for a function as defined in Eq. (1) of the form *y*=*a*(1− exp(−*b* *x*))+*c* has been applied to the data, where *a*, *b* and *c* are fitting parameters. The coefficient of determination shows poor correlation with the data, with *R* ^2^ values of 3.7·10^−3^ and −1.03·10^−4^ for CCVJ and DCVJ, respectively. The results do not show a correlation between stirring rate and intensity.

**Fig. 7:**
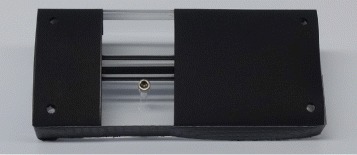
Experimental Setup for Continuous Excitation. The channel has been covered with black paper to define the starting point of isomerization. Flow from *left* to *right*

### Poiseuille flow

For the flow chamber E, two different experiments were conducted. On the one hand, single shot experiments were conducted to study the emission of CCVJ with a high concentration of E isomer. On the other hand, continuous excitation was applied for comparison with the other flow chambers.

#### Single shot excitation

The experiment was set up as described in “Single shot excitation” in the “Methods” section. The fluid was held in the dark to guarantee a high concentration of E isomer. The laser sheet was aligned parallel to the flow direction at the center of the channel. After establishment of the flow, the fluorescence of a single laser shot was acquired with the camera. For each acquisition cycle, 20 images were acquired and averaged.

The computed intensity change did not reveal a dependency on the established flow. The result can be described statistically by a Gaussian shape with a deviation of 1 %. Results are not shown here, but the raw data is available in a repository [18].

#### Continuous excitation

**Fig. 8:**
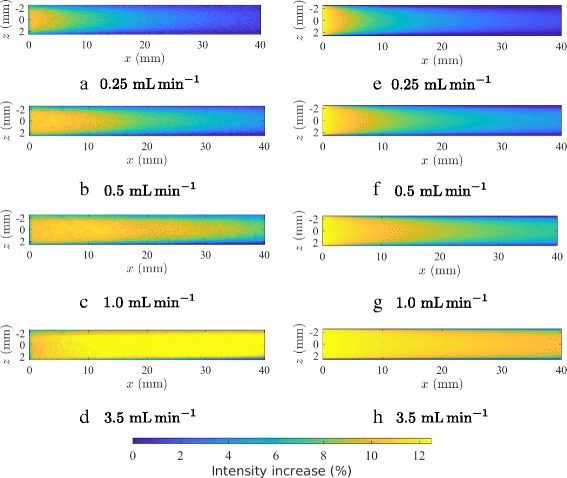
Intensity increase of Poiseuille flow in rectangular channel. Experimental data (subfigures (**a**) through (**d**)) and analytical model ((**e**) through (**h**))

For these measurements, a region of interest was defined 50 mm downstream of the inlet for a length of 40 mm. The channel upstream of that region was shielded with black paper to avoid exposure of the fluid to the LED light. The covered flow chamber is shown in Fig. 7. The setup was established as described in “Continuous excitation” in the “Methods” section. The flow images were acquired after establishment of a flow. The results are shown in Fig. 8(a) to (d). The origin of the coordinate system (*x*=0) has been defined at the end of the cover, where the fluid is exposed to illumination the first time. Intensity increases are observable, depending on the flow velocity and the position in the channel, with the highest increases in the center of the channel.

Furthermore, the intensity distributions were integrated in the flow direction *x* for comparison. A three pixel wide one-dimensional median filter was applied to the final result of the integration. The result can be found in Fig. 10.

**Fig. 9:**
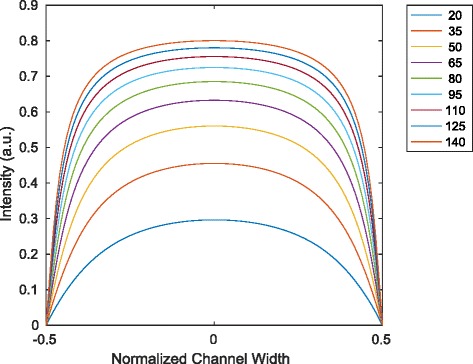
Dependence of fluorescence signal on *v*_0_. The results are shown for various *α* under the assumption of a rectangular profile (from model in Eq. (23))

**Fig. 10:**
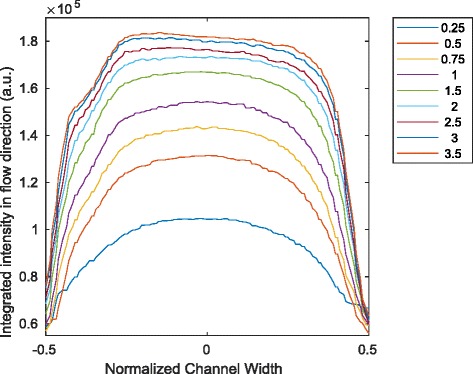
Integrated intensities of Poiseuille flow. The experimental results of Fig. 8 are integrated in the flow direction. The legend represents the volumetric flow rate in mL min^-1^

#### Analytical result of rectangular flow

Results of the analytical model described in “Intensity decay in a rectangular Poiseuille flow” in the “Methods” section can be found in Fig. 8 (e) to (h). The input parameters were set to *I*_*E*,0_/*I*_*base*_=0.125 and *t*_*decay*_ = 50 s. While the relation *I*_*E*,0_/*I*_*base*_ has been set according to the result of the maximal intensity change of the experiment shown in Fig. 8(d), the decay time is within a range of values which were investigated independently with resting fluid and the LED as illumination source to simulate the illumination conditions as close as possible. A parametric fit for the fluorescence lifetime based on the measured spatio-temporal intensity decay yields values close to the independently determined value. It has to be assumed that the decay time depends on the illumination source and the experimental setup [4, 5].

#### Fluorescence signal of Poiseuille flow in flow direction

Given the solution of the rectangular Poiseuille flow in (3) an integration in the *y* direction yields the average velocity

22 $$\begin{array}{*{20}l} \bar{u}(z) &= \frac{1}{h}\int\limits_{-\frac{h}{2}}^{\frac{h}{2}} u(y,z) dy' \end{array} $$

23 $$\begin{array}{*{20}l} &= -\frac{1}{\eta}\frac{\partial p}{\partial x} h^{2}\left[\frac{1}{12} - \frac{8}{s_{n}} \sum_{n=1}^{\infty} \frac{(-1)^{n}}{s_{n}^{3}}\frac{\cosh(s_{n}K\frac{z}{w})}{\cosh\left(s_{n}K\frac{1}{2}\right)}\right.\\ &\qquad\qquad\qquad\times\left.\sin\left(0.5s_{n}\right){\vphantom{\sum_{n=1}^{\infty}}}\right]  \end{array} $$

The result can be applied to the result for the fluorescence intensity in (17) with the linearity assumption described in (18). With the substitution $\alpha = -\frac {1}{\eta }\frac {\partial p}{\partial x}$ an intensity signal can be computed. Results are presented in Fig. 9 for various *α* and 99 terms in the sum. In the case of the fluorescence signal, the motivation is to compare results with Haidekker et al. [3] and their presented experiment of a fluorescence signal recorded by a fiber. In their paper, no intensity changes as expressed in Eqs. (1) or (2) were evaluated.

For comparison, Fig. 10 shows the integrated results of intensity from Fig. 8.

### Chamber Flows

Experiments with continuous excitation were conducted with the different flow chambers A to D. The experiments were set up with the same parameters as described by Mustafic et al. [4]. The findings of the intensity changes in the flow fields were verified. The results are not shown here, but raw data is available in a data repository [18].

#### Reversed flow in chamber B

In addition to the verification of Mustafic et al. [4], the flow in chamber B was reversed. Since this is the only chamber with an asymmetric setup, it is possible to investigate the present hypotheses.

Except for the change of inlet and outlet, all other experimental parameters were set as in the preceding experiments. The results for different volume flow rates are shown in Fig. 13.

## Discussion

### Stirring

In the present results, no correlation between stirring rate and intensity was found. The results for CCVJ do not differ from those of DCVJ. This outcome contrasts preceding findings and raises the question why there is a discrepancy in the results [3–6].

The setup was deliberately different from experiments in a spectrofluorometer. The full illumination of the laboratory bottle was chosen to achieve a stable isomer distribution. The illumination source should have no influence on the results, because both isomers occur in a chemical equilibrium. This is different to experiments with a laser beam passing through a sample, were isomerization takes place.

Usually, spectrofluorometers have a single laser light beam for illumination of the test section. Since no adaptation of the setup is reported for the other stirring and pumping experiments, we assume the optical setup was not changed [3, 4]. In a spectrofluorometer a small cuvette is filled with a sample. This sample is then illuminated and the fluorescence signal of the sample recorded. The devices used in the aforementioned experiments apply a focused light beam for the excitation of the sample. Thus, in an experiment, just a small fraction of the sample solution is illuminated by laser light. Since the rate constant depends on the illumination intensity, this leads to a fast isomerization to Z isomer. A stirring of the sample solution then results in a continuous provision of E isomer and a higher measured intensity.

For pumping experiments within the spectrofluorometer as presented by Rumble et al. [5] the same explanation can be referred to. The results presented in Fig. 2 in their publication show high intensity peaks during the operation of the pump. There is a visible saturation effect for higher pumping speeds. This can be explained with photoisomerization. From a fluid dynamical perspective, the advection of E isomer into the laser beam is higher than the rate constant of isomerization, which limits the maximal intensity. Furthermore, an intensity decay is observable after stopping the pump. Under the assumption of an immediate stop of the syringe pump and a closed flow cycle, no long-term movement of the fluid should be observable. The excitation is realized with a localized laser beam, but the fluorescence acquisition captures not only the laser beam area. In the stopped setup the fluid within the laser beam should isomerize rapidly to the non-fluorescent state. Under the assumption of a dependency of the rate time constant on the illumination intensity, one has to take into account scattered light. The remaining sample solution experiences illumination from scattered light as well, but at a different order of magnitude. This results in a significantly lower rate constant and a slower intensity decay.

As conclusion we suppose that the measured intensity changes of experiments [3–6] depended on the experimental setup and especially the use of a spectrofluorometer and not a velocity or shear dependency of CCVJ. Additional experiments with adapted optics in a spectrofluorometer could test our hypothesis.

### Poiseuille flow

For the Poiseuille flow two experiments and two analytical models were presented. The importance of the Poiseuille flow for the current debate is directly related to suggested empirical Eqs. (1) and (2).

First, the experiment with single shot excitation does not show a correlation between flow velocity or shear and the luminescence intensity. Even though a high amount of E isomer was present in the sample solution, no intensity change could be observed above the noise level of the experimental setup.

Second, the experiment with a continuous excitation source provides a significant increase in intensity. This increase depends on the volume flow rate and on the measurement position in the channel. More importantly, the result is in contradiction to the assumption of a shear sensitivity, since the shear profile in a Poiseuille flow does not match the measured intensity changes. The solution for the shear in a plane Poiseille flow is given by

24 $$ \tau = \eta\frac{\partial u}{\partial z} = z\frac{\partial p}{\partial x}  $$

with the coordinate system as defined before, in particular with its origin in the center of the channel ([19], p. 301). It can be seen that the intensity increase of the experiments does not follow the shear magnitude for a Newtonian fluid. The channel in the experiment is of rectangular, not of quadratic shape, but the result of the shear will qualitatively be the same with no shear in the center of the channel and its maximum at the walls.

Third, the decay model presented in “Intensity decay in a rectangular Poiseuille flow” subsection agrees qualitatively with the experimental results, as can be seen in Fig. 8, comparing experimental results (a) to (d) with model results (e) to (h).

Fourth, the second analytical model with assumptions from reaction kinetics and fluid dynamics provides a consistent explanation for both the comparison with the experiments conducted in this work and with those presented by Haidekker et al. [3] The agreement of the results in Figs. 9 and 10 is highly indicative.

Thus the results of the continuous excitation experiment contradict the supposition of a shear sensitivity of CCVJ. On the other hand comparison of the experiment with two different theoretical models shows a good agreement with the hypothesis of photoisomerization.

### Flow chamber B with reversed flow

**Fig. 11:**
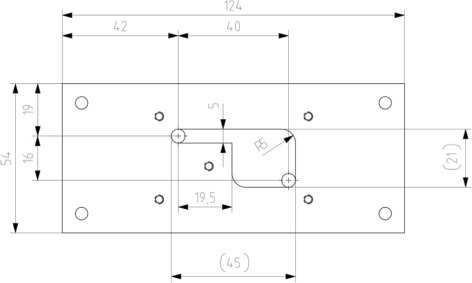
Dimensions of Chamber B. The depth of the chamber is 3 mm

Mustafic et al. [4] examined a series of flow chambers to support the hypothesis of flow velocity or shear sensitivity. In Fig. 11 the dimensions and principle setup of the chamber are shown. It is equivalent to the chamber presented by Mustafic et al. [4].

**Fig. 12:**
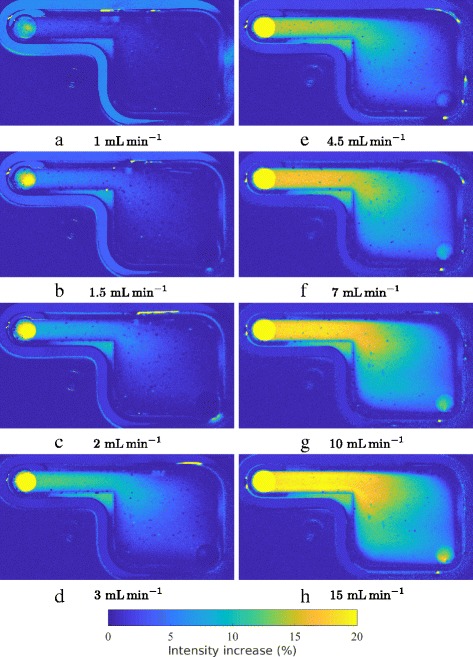
Intensity increase of original flow at different flow rates (**a** to **h**) in chamber B. The inlet of the flow is in the north-west of the chamber

**Fig. 13:**
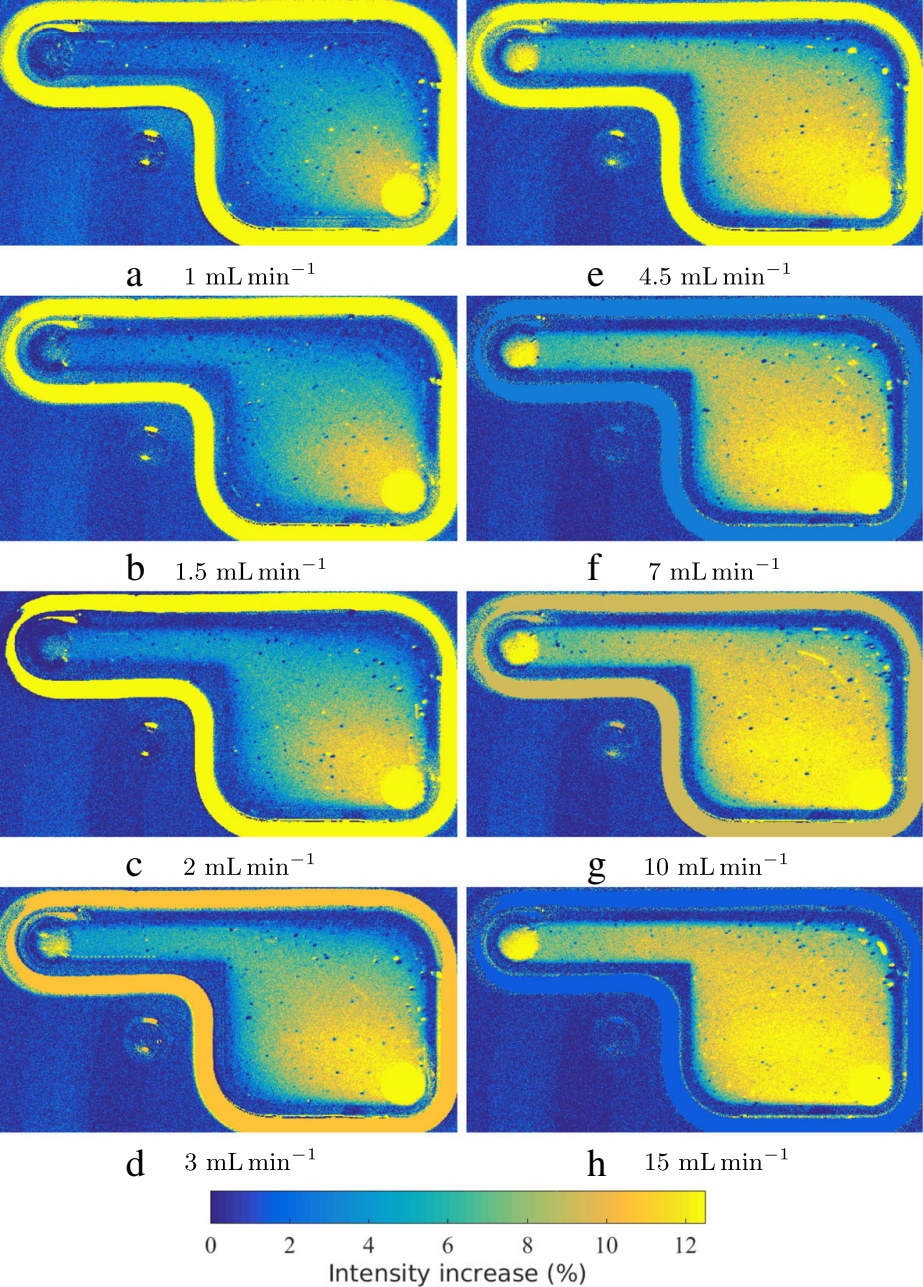
Intensity increase of reversed flow at different flow rates (**a** to **h**) in chamber B. The inlet of the flow is in the south-east of the chamber

Regarding the velocity field in the chambers, a contradiction to the velocity hypothesis can be found at the inlets and outlets of the chambers. The intensity increase can only be found at the inlets of the chambers. For comparison, Fig. 12 shows the result of the flow from left to right. The intensity increase is observable at the inlet in the small region of the chamber. In contrast, the result of the reversed flow is shown in Fig. 13. No intensity increase is observed in the small region in the upper left part of the chamber. With regard to the supposition of isomerization, this result could be explained with a solution of high E isomer concentration entering the chamber and reacting to Z isomer due to illumination. In the images fluorescence of the rubber sealing is observable as well which does not influence the overall results.

With regard to the reversed flow in chamber B, the isomer hypothesis can be confirmed. If the intensity had depended on the velocity, an increase would have been measured in the upper left narrow channel. Instead, the results show a plume at the new inlet with a dependency on the volume flow rate.

## Conclusions

The present study tested three hypotheses regarding the behavior of CCVJ in fluid flows. The first hypothesis relates to a velocity sensitivity, the second to a shear sensitivity, and the third introduces photoisomerization as a cause for the intensity changes.

Investigations were performed considering three different aspects regarding fluid flow: Stirring of a CCVJ sample solution, a Poiseuille flow, and flow chambers according to Mustafic et al. [4]

The strongest indication of a connection between velocity or shear and CCVJ was presented in stirring and pumping experiments in a spectrofluorometer [3–6]. Comparable results without a spectrofluorometer were presented indicating that the measured intensity increase is related to the measurement setup and not a direct physical effect in the CCVJ-doped fluid flow. With a complete and permanent illumination of the solution sample, no correlation between stirring rate and intensity were found.

A Poiseuille flow was examined in two experiments and two theoretical models considering effects of photoisomerization were derived. The experiment with single shot excitation, which effectively eliminates photoisomerization, does not reveal any intensity change. On the other hand, the isomerization dependent experiment with continuous excitation establishes an intensity increase. This increase is in good agreement with the two presented theoretical models as well as the results of Poiseuille flow measurements with an optical fiber by Haidekker et al. [3] This agreement suggests isomerization as explanation for the intensity increase. The hypothesis of a shear sensitivity has to be rejected because the intensity change pattern does not agree with the shear profile present in a Poiseuille flow.

The results of the flow chamber experiments are in good agreement with Mustafic et al. [4]. The reverse flow in chamber B indicates again an isomerization effect to be responsible for the observed intensity changes.

While not examined in detail, photobleaching does not seem to occur with CCVJ. During the repeated experiments the same sample solution was used. The stirring experiments were conducted repeatedly. With full illumination of the sample solution for several hours, no bleaching were observable.

In conclusion, no experimental evidence of a flow-related effect next to photoisomerization has been found. The hypothesis of a shear sensitivity can be dismissed based on results of the experiment with continuous excitation in a Poiseuille flow. The experiment with single-shot excitation does not reveal a flow dependency even though the E isomer of CCVJ is assumed to have a significantly higher concentration than the Z isomer. If there is a flow dependence present, it was not observable within the sensitivity limits of the experimental setups. The only finding supporting an additional effect can be the comparison of the Poiseuille flow with the model described in subsection “Intensity decay in a rectangular Poiseuille flow”. But the discrepancies can be explained with an uncertainty regarding the chosen parameter values for the decay time and the initial intensity. An experiment of the reversed flow in chamber B does not support a velocity sensitivity. If such a dependence of fluorescence were present, a change of intensity in the narrow outlet would be observable.

## Abbreviations

CCVJ: 9-(2-Carboxy-2-cyanovinyl)julolidine
CFD: Computational fluid dynamics
DCVJ: 9-(2,2-Dicyanovinyl)julolidine
DMSO: Dimethyl sulfoxide
LED: Light emitting diode
PIV: Particle image velocimetry
ROI: Region of interest
